# Transparent inorganic multicolour displays enabled by zinc-based electrochromic devices

**DOI:** 10.1038/s41377-020-00366-9

**Published:** 2020-07-14

**Authors:** Wu Zhang, Haizeng Li, William W. Yu, Abdulhakem Y. Elezzabi

**Affiliations:** 1 Ultrafast Optics and Nanophotonics Laboratory, Department of Electrical and Computer Engineering, University of Alberta, Edmonton, Alberta T6G 2V4 Canada; 2 Department of Chemistry and Physics, Louisiana State University, Shreveport, LA 71115 USA

**Keywords:** Nanowires, Displays

## Abstract

Electrochromic displays have been the subject of extensive research as a promising colour display technology. The current state-of-the-art inorganic multicolour electrochromic displays utilize nanocavity structures that sacrifice transparency and thus limit their diverse applications. Herein, we demonstrate a transparent inorganic multicolour display platform based on Zn-based electrochromic devices. These devices enable independent operation of top and bottom electrochromic electrodes, thus providing additional configuration flexibility of the devices through the utilization of dual electrochromic layers under the same or different colour states. Zn–sodium vanadium oxide (Zn–SVO) electrochromic displays were assembled by sandwiching Zn between two SVO electrodes, and they could be reversibly switched between multiple colours (orange, amber, yellow, brown, chartreuse and green) while preserving a high optical transparency. These Zn–SVO electrochromic displays represent the most colourful transparent inorganic-based electrochromic displays to date. In addition, the Zn–SVO electrochromic displays possess an open-circuit potential (OCP) of 1.56 V, which enables a self-colouration behaviour and compelling energy retrieval functionality. This study presents a new concept integrating high transparency and high energy efficiency for inorganic multicolour displays.

## Electrochromic displays: superior colour

Transparent electrochromic displays with a richer colour palette are now possible. Electrochromic displays, which change their colour due to electrochemistry, are receiving interest due to their low power consumption but a lack of colours is problematic. Wu Zhang and coworkers from the University of Alberta in Canada and Louisiana State University in the USA fabricated multicolour displays using sodium ion stabilized vanadium oxide nanorods (Zn-SVO) as the electrochromic material. Two layers of this material, separated by a layer of zinc and a gel electrolyte were sandwiched between glass coated with indium tin oxide. Application of a small voltage causes the SVO film to exhibit a reversible switch between orange, yellow and green. The use of two independently-controlled films in the display provides a broader range of colours (orange, amber, yellow, brown, chartreuse and green).

## Introduction

Due to their zero energy consumption while maintaining either an optically transparent or coloured state, electrochromic devices have attracted increased attention for various applications, including smart windows, displays and colour-tuneable optical elements^1–3^. In particular, multicolour electrochromic displays are one of the most versatile applications because they can retain their coloured states without the need to supply electrical power. While electrochromic displays based on organic molecules^4^, polymers^5^ and metal-organic frameworks^6^ have demonstrated multicolour characteristics, these materials exhibit inferior thermal and chemical stabilities compared to their inorganic electrochromic counterparts. These shortcomings seriously hinder their real-world applications and potential commercialization^7^. Inorganic multicolour electrochromic displays are regarded as a paradigm shift in the field of electrochromic displays.

Recent demonstrations of inorganic multicolour electrochromic displays achieved multicolour functionality from a monochromatic WO_3_ film by incorporating either a photonic Fabry–Perot nanocavity^7^ or a plasmochromic metal–insulator–nanohole cavity^8^. These platforms enabled multicolour addressing in reflective mode. For a wide scope of applications, it is highly desirable to have a device configuration possessing high optical transparency while also expressing coloured states^9–11^. Notably, in terms of energy efficiency, the aforementioned reflective-mode devices consume electrical energy since an external voltage is needed to trigger the colouration/bleaching processes. Although this field is still in its infancy, electrical energy recovery from multicolour electrochromic displays will render such a platform highly energy efficient, especially for large-area displays.

**Fig. 1:**
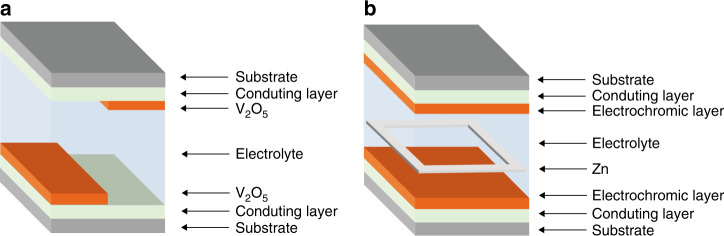
Schematic illustrations of two different types of electrochromic devices. **a** Conventional electrochromic device using V_2_O_5_ films for both electrochromic and ion storage electrodes. Note that in this configuration, there is no overlap between the electrochromic layers; as such, no colour overlay effect and no broadened colour palette are possible. **b** Zn-based electrochromic device using a Zn-foil anode frame. This configuration facilitates colour overlay for the two segments of electrochromic electrodes.

In addition to nanocavity-based inorganic multicolour displays, a colour overlay strategy can be a simpler approach to broaden the colour palette of an inorganic electrochromic device by superimposing layers of different colours (i.e., colour overlay). Vanadium oxide (V_2_O_5_) is regarded as the most promising inorganic material for multicolour electrochromic displays^12–14^. To the best of our knowledge, there are only three colours (yellow ⇄ green ⇄ blue) that can be achieved by vanadium oxides using the conventional electrochromic device configuration^13,15^. Simultaneous colouration of the counter layer when operating a conventional electrochromic device restricts the colour overlay effects. Since V_2_O_5_ electrodes can serve as both electrochromic and counter layers^13^, the configuration illustrated in Fig. 1a can be configured to eliminate the simultaneous colouration effect of the counter V_2_O_5_ electrode. However, only three colours can be realized under different redox states since the top and bottom V_2_O_5_ electrodes can only colour under inverse redox states when operating the device.

Recently, we demonstrated a Zn-based electrochromic device consisting of a thin Zn-foil anode sandwiched between two electrochromic electrodes (e.g., WO_3_, PB) (Fig. 1b)^16,17^. These devices show great potential for smart windows due to the monochromatic characters of WO_3_ and PB films. Therefore, we focused on this configuration to reduce the transmission of the tinted state by combining the simultaneous colouration of both electrochromic electrodes. However, the development of Zn-based electrochromic devices with independent colour activation of top and bottom electrochromic electrodes is still lacking and needs to be further explored. Specifically, the top and bottom electrochromic electrodes can be independently addressed under the same or different redox states. As such, the colour overlay effect can greatly broaden the colour palette. Moreover, the operation of traditional electrochromic devices requires external voltages to trigger the colouration/bleaching processes, which makes the traditional electrochromic devices far from a net-zero energy consumption technology. Remarkably, this novel Zn-based electrochromic device architecture is able to self-colour via its built-in battery power without external energy input, thus enabling partial retrieval of the electrical energy consumed during the bleaching process. In other words, this Zn-based electrochromic device operates as a secondary device where the device can be bleached during the charging process and spontaneously coloured during the discharging process. Hence, to date, this Zn-based electrochromic device represents the most promising energy-efficient platform for transparent inorganic multicolour display applications.

In this work, we demonstrate a novel concept for transparent inorganic multicolour electrochromic displays by employing sodium ion stabilized vanadium oxide (SVO) nanorods as the electrochromic material. Although the intercalation of sodium ions was shown to improve the electrical conductivity of SVO in zinc-ion batteries^18^, to date, there is no report on the utilization of SVO for electrochromic devices. The current state-of-the-art vanadium oxide-based electrochromic display research has focused on developing nanostructured vanadium oxides (e.g., V_2_O_3_, V_3_O_7_, V_2_O_5_)^13,19,20^ without paying attention to potential electrochromic materials such as SVO. The SVO nanorods are compatible with a simple bar-coating method for fabricating electrochromic films when mixed with cellulose. Due to the oxidation nature of SVO^21^, the added cellulose can be fully decomposed under a low temperature (200 °C) to prevent its influence on the conductivity.

The performance of a single SVO electrode was measured via a Zn–SVO electrochromic system configured with a SVO cathode, a Zn anode, and a 1 M ZnSO_4_ electrolyte. We show that the SVO film exhibits reversible multicolour switching (orange ⇄ yellow ⇄ green) during the Zn^2+^ insertion (self-colouring/discharging) and extraction (bleaching/charging). The orange colour is attributed to the presence of sodium in SVO. With a 2 V bias, the SVO film colours orange and exhibits a high optical transmission of ~71% at 632.8 nm. Both the optical transmission and the colour state can be tuned by applying different voltages, with the highest optical transmittance contrast of 21% at 632.8 nm. Such a SVO film features a rapid self-colouration time of 7.8 s and switching times of 12.6/25.4 s for the colouration/bleaching processes. Remarkably, the SVO film in a Zn–SVO electrochromic platform not only eliminates the need for an applied voltage to trigger the colouration process but also retrieves 14.8 mWh m^−2^ from the energy consumed during the bleaching process. Notably, this novel energy retrieval functionality offers an energy efficiency advantage over conventional electrochromic displays. By taking advantage of the three-colour (orange ⇄ yellow ⇄ green) electrochromic response of the SVO film, an electrochromic display was constructed by sandwiching zinc foil between two SVO electrodes (schematically shown in Fig. 1b). The Zn–SVO electrochromic display possesses an open-circuit potential (OCP) of 1.56 V, which enables a self-colouration behaviour and energy retrieval functionality. Using the colour overlay effect, we demonstrated a device displaying six colours (i.e., orange, amber, yellow, brown, chartreuse and green). This is the first demonstration of transparent inorganic multicolour displays having more than three colours. Such multicolour features and energy retrieval functionality are expected to be significant catalysts in accelerating the development of future energy-efficient electrochromic displays.

## Results

### Characterization of SVO nanorods

**Fig. 2:**
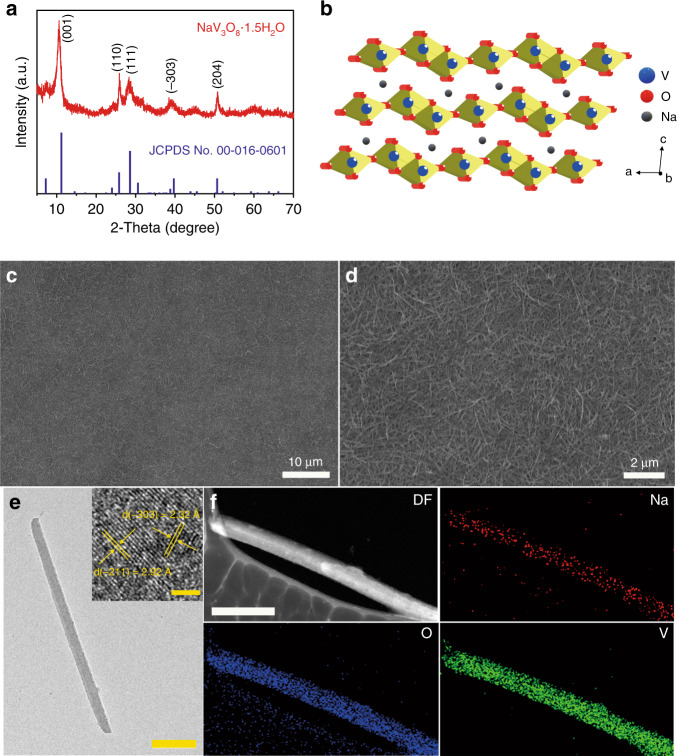
Characterization of SVO nanorods. **a** XRD pattern of the SVO nanorods. **b** Crystal structure of the SVO nanorods. **c**, **d** FESEM images of the SVO nanorods. **e** Bright-field (BF) TEM image of a single SVO nanorod (scale bar: 100 nm). Inset: high-resolution TEM image of a SVO nanorod depicting the lattice planes (scale bar: 2 nm). **f** Dark-field (DF) TEM image of a SVO nanorod and the corresponding elemental mapping images of Na, O, and V (scale bar: 200 nm).

The SVO nanorods were prepared at room temperature using a high yield facile liquid–solid stirring method (see the “Materials and methods” section). To analyse the phase composition of the as-synthesized SVO, the suspension was dried at room temperature and characterized by powder X-ray diffraction (XRD). As shown in Fig. 2a, the diffraction peaks of SVO can be indexed to the monoclinic NaV_3_O_8_·1.5H_2_O (JCPDS No. 00-016-0601) phase. The hydrated sodium ions, inserted between the V_3_O_8_ layers, act as pillars to stabilize the layered structure (Fig. 2b). Figure 2c, d shows field emission scanning electron microscopy (FESEM) image of the SVO, depicting a homogeneous nanorod morphology. The SVO nanorods range from 0.5 to 2.0 μm in length and 20 to 60 nm in diameter (Supplementary Fig. S1 and Supplementary Table S1). The transmission electron microscopy (TEM) image (Fig. 2e) also affirms the high aspect ratio of the SVO nanorod morphology. The high-resolution TEM image (Fig. 2e inset) shows crystalline lattice spacings of 2.27 and 2.92 Å corresponding to the (−303) and (−211) crystal planes, respectively. The peak of the (−211) crystal plane observed in the HRTEM image is covered by the (111) plane peak in the XRD pattern. As the standard crystal plane peaks from JCPDS No. 00-016-0601 in Fig. 2a show, the (−211) crystal plane peak is close to the highly preferred (111) crystal plane peak with a much lower peak intensity. Thus, the (−211) crystal plane peak would be covered by the (111) plane peak in the XRD pattern. The TEM elemental mapping images in Fig. 2f illustrate the homogeneous distributions of Na, V and O elements in the SVO nanorods, thus confirming that the sodium ions have been intercalated into the V_3_O_8_ interlayers.

### Fabrication of SVO electrodes via the bar-coating method

**Fig. 3:**
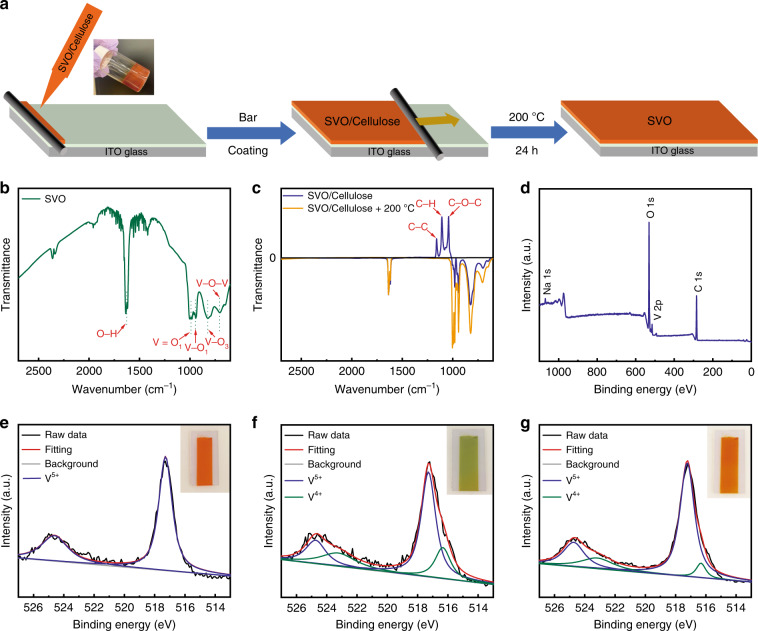
Fabrication of SVO films via the bar-coating method. **a** Schematic diagram of the bar-coating process. The inset shows a digital photograph of the SVO/cellulose paste. FTIR spectra of **b** SVO, **c** SVO/cellulose and the annealed SVO film. **d** XPS survey spectrum of the as-prepared SVO/cellulose film. V 2p core-level XPS spectrum of the bar-coated SVO/cellulose film under different annealing states: **e** as-prepared, **f** annealed for 30 min and **g** annealed for 24 h. The insets in (**e**–**g**) illustrate the colour of the corresponding films.

The facile bar-coating method is considered a promising candidate to replace the common vacuum‐processing techniques due to its great potential for large-scale, rapid and inexpensive manufacturing^22^. Notwithstanding, the fabrication of electrochromic films via the facile bar-coating process requires a high-quality electrochromic paste. We therefore prepared a SVO paste via the addition of water-soluble and eco-friendly cellulose (see the “Materials and methods” section for details). Cellulose can be uniformly dispersed in the SVO suspension due to its water-soluble property, while it can be easily decomposed at a low temperature due to the oxidation nature of SVO. Complete decomposition of cellulose prevents possible residual dispersion in the aqueous electrolyte system, which may affect the electrical conductivity of the SVO films. Figure 3a illustrates a schematic diagram of the bar-coating process that comprises three steps: (i) load the SVO/cellulose paste on one end of the substrate, (ii) spread the SVO/cellulose paste over the substrate through horizontal sliding of a coating bar and (iii) anneal the bar-coated film at 200 °C for 24 h in air to decompose the cellulose. Using this facile bar-coating method, we obtained ~800-nm thick SVO films as measured by an Alpha-Step probe (IQ-W1-040).

To confirm that the cellulose was fully decomposed from the film after heating, Fourier transform infrared spectroscopy (FTIR) was employed to characterize the SVO nanorods, SVO/cellulose paste and annealed SVO films. As shown in Fig. 3b, the absorption bands at ~1633 and at 1003, 945, 830 and 714 cm^−1^ in the SVO film spectrum are assigned to O–H vibrations of water and to stretching vibrations of V=O_1_, V–O_1_, V–O_3_ and V–O–V^23,24^, respectively. Figure 3c shows the FTIR spectra of the as-prepared SVO/cellulose paste and the annealed SVO films. To distinctly reveal the cellulose absorption bands, the FTIR spectra were corrected based on the SVO nanorod spectrum (Fig. 3b). In the spectrum of the as-prepared SVO/cellulose film before annealing, the absorption bands at approximately 1158, 1106 and 1042 cm^−1^ are assigned to the stretching vibration of C–C, C–H in-plane bending and stretching vibration of C–O–C, respectively. These absorption bands originate from cellulose, confirming the existence of cellulose in such films^25,26^. After annealing at 200 °C for 24 h, no functional groups of cellulose are found in the spectrum (Fig. 3c), indicating complete decomposition of cellulose in the annealed SVO film.

Ex situ X-ray photoelectron spectroscopy (XPS) measurements were carried out to investigate the valence states of SVO during the heating process. As shown in Fig. 3d, the XPS survey spectrum of the as-prepared SVO/cellulose film indicates the presence of Na, V, C and O elements. The intense peaks of C and O elements are attributed to the presence of cellulose^27^. Figure 3e–g depicts the high-resolution V 2p core-level XPS spectra of the bar-coated SVO/cellulose film under different annealing conditions (as-prepared, annealed for 30 min and annealed for 24 h). The most intense doublet peaks, located at 517.2 and 524.6 eV, are assigned to V^5+^^20,28^. The other pair of peaks centred at 516.2 and 523.2 eV correspond to V^4+^. In Fig. 3e, only V^5+^ peaks exist in the spectrum of the as-prepared film, which is consistent with the valence state of V in SVO. After annealing the bar-coated film for 30 min (Fig. 3f), V^4+^ peaks appear, and the atomic ratio of V^4+^/V^5+^ increases to 0.41 (Supplementary Table S2). The presence of the V^4+^ peaks indicates that SVO serves as an oxidizer to oxidize the cellulose, leading to decomposition of the cellulose at a low temperature. After 24 h of annealing, the spectrum, shown in Fig. 3g, shows that the V^4+^/V^5+^ atomic ratio decreases to 0.12 (Supplementary Table S2). This result affirms that the cellulose was fully decomposed and that the reduced V^4+^ was oxidized to V^5+^ by annealing. Similar to the electrochromic effect, the valence state evolution of V induces a colour variation of the bar-coated film. As presented in the insets in Fig. 3e–g, the bar-coated film displays colour switching (orange → light green → orange) during the annealing process. The light green colour is attributed to the presence of V^4+ 20^. Supplementary Fig. S2 shows FESEM images of the SVO film after the annealing process. The homogenous nanorod morphology is consistent with the morphology of the as-prepared SVO nanorods (i.e., Fig. 2c, d).

### Electrochromic performance of the SVO electrodes

**Fig. 4:**
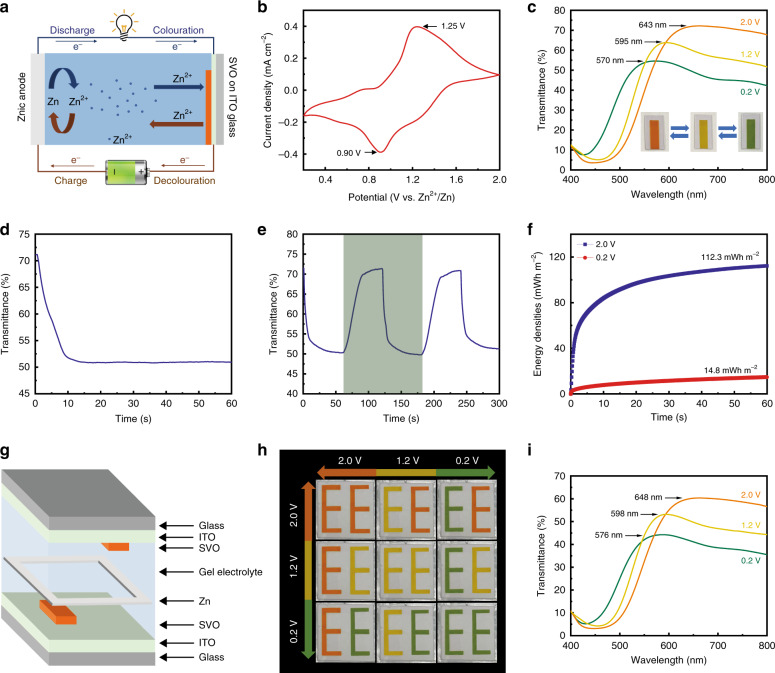
Electrochromic performance of the SVO electrode. **a** Schematic illustration of a Zn–SVO electrochromic display platform. **b** CV curve of the SVO electrode at a scan rate of 5 mV s^−1^ over a voltage range of 0.2–2.0 V. **c** Visible-near infrared transmittance spectra of the SVO electrode under different voltages for 60 s: green (0.2 V), yellow (1.2 V) and orange (2.0 V). Inset: corresponding photographs of the SVO electrodes under different voltages. **d** In situ self-colouring process (spontaneous colour switching from orange to green) of the SVO film. **e** Dynamic test of the SVO film at 632.8 nm in the 0.2–2.0 V window. **f** Round-trip energy density comparison of the SVO electrode during the selected cycle (dark-green region in (**e**)) of the dynamic test. **g** Schematic illustration of a large-scale Zn–SVO electrochromic display having three intrinsic colours. **h** Digital photographs of the large-scale Zn–SVO display under different voltage bias conditions. **i** Visible-near infrared transmittance spectra of the large-scale Zn–SVO display under different voltages: green (0.2 V), yellow (1.2 V) and orange (2.0 V).

The electrochromic performance of the SVO electrodes was characterized via a two-electrode configuration. Here, zinc foil and a SVO electrode were used as the anode and cathode, respectively, and 1 M ZnSO_4_ was used as the electrolyte solution. The choice of the ZnSO_4_ solution as the electrolyte is supported by the fact that the SVO electrode is more electrochemically active towards Zn^2+^ (Supplementary Fig. S3). Figure 4a illustrates a schematic diagram of the working principle of the Zn–SVO electrochromic display. The redox potential difference between the zinc foil and the SVO electrode provides the driving force that activates oxidation of Zn and reduction of the SVO film. Such a process is similar to the discharging process of a secondary battery. During the discharging process, the redox potential difference induces oxidation of the Zn anode (Zn → Zn^2+^) and intercalation of Zn^2+^ into the SVO cathode. Thus, this discharging process triggers a self-colouration behaviour. In the charging process, the applied external voltage induces deintercalation of Zn^2+^ from the SVO cathode and reduction of Zn^2+^ (Zn^2+^ → Zn). The charging process, therefore, triggers a bleaching behaviour.

As depicted in Fig. 4b, the pair of reduction and oxidation peaks (at ~0.90/1.25 V) observed in the cyclic voltammetry (CV) curve indicate a single-step intercalation and extraction process of Zn^2+^ through the SVO lattice. The ex situ XPS analysis, shown in Supplementary Fig. S4, intrinsically supports the conclusion that the spontaneous colour switching behaviour of the SVO electrode is attributed to the intercalation of Zn^2+^ ions. Figure 4c shows the change in the optical transmittance spectra of the SVO electrode under different applied voltages. Clearly, different colours are realized under different applied voltages. The transmission peak blueshifts as the applied voltage decreases. The processes of Zn^2+^ insertion (self-colouring) and extraction (bleaching) allow the SVO electrode to exhibit reversible colour switching (orange ⇄ yellow ⇄ green, inset in Fig. 4c). Remarkably, the SVO electrode displays different colours while still preserving its high optical transparency of >50%. This key feature can potentially provide access to new applications that are unattainable with reflective-mode electrochromic displays^7,8^. The gradual colour switching from orange to green results in a 73 nm blueshift of the transmission peak from 643 to 570 nm, while achieving a 21% optical contrast at 632.8 nm (without subtracting the transmittance loss of the ITO-coated glass). This gradual colour switching is attributed to the cathodic colouration effect of the SVO, which is similar to that for our previously reported V_3_O_7_ electrodes^20^. To further investigate the self-colouration behaviour (spontaneous colour switching from orange to green) of this SVO electrode, the self-colouration process was monitored by applying 0 V for 60 s (equivalent to connecting the Zn and SVO electrodes together, Fig. 4d). The self-colouration time (spontaneous colour switching time), defined as the time required to achieve 90% of the maximum optical contrast^29^, was measured to be 7.8 s. Furthermore, the dynamic transmittance characteristics of the SVO electrode were tested within the applied voltage window between 0.2 and 2.0 V. As shown in Fig. 4e, the response times are calculated to be 12.6 s for colouration (green) and 25.4 s for bleaching (orange). Notably, this Zn-based SVO electrochromic display eliminates the need for electrical energy to trigger the colouration process, thus making it more energy efficient than existing electrochromic displays^7,8^.

Remarkably, the self-colouration (spontaneous colour switching) process enables partial retrieval of the energy expended during the bleaching process. An important metric that characterizes our Zn-based SVO electrochromic display performance is the round-trip net energy consumption. The energy densities of both the colouration process and the bleaching process during a selected round-trip cycle in Fig. 4e are compared and presented in Fig. 4f. Here, the SVO electrode recovers 14.8 mWh m^−2^ from 112.3 mWh m^−2^ in a selected round-trip cycle (i.e., the round-trip net energy consumption is 97.5 mWh m^−2^). While the amount of retrieved energy can be further increased by operating at higher discharge voltages, such a low discharge voltage is found to be adequate for counterbalancing the interplay between the fast switching time requirement and the energy retrieval capability. Along with the dynamic switching shown in Fig. 4e, the colouration efficiency (CE) of the SVO electrode is calculated to be 61.2 cm^2^ C^−1^ (Supplementary Fig. S5). This CE value is higher than the values reported for Li^+^-based vanadium oxide electrochromic films^30,31^, indicating that Zn^2+^ is efficient and promising in SVO-based electrochromic displays. Moreover, Supplementary Fig. S6 shows that the cycling performance of the Zn–SVO electrochromic display is higher than those in published works^20,32,33^, where it maintains 51% of its initial capacity and retains ~62% of the optical contrast after 1000 CV cycles.

The bar-coating method, as expected, can be used to fabricate large-area electrochromic displays with patterns. A 100-cm^2^ display having three intrinsic colours was constructed to demonstrate the scalability of the bar-coated SVO electrode (Fig. 4g). Such a configuration enables independent operation of the top and bottom SVO electrodes, thus providing additional configuration flexibility in displaying patterns (i.e., different colour arrays) compared to the device illustrated in Fig. 1a. The colours of the two letters “EE”, as depicted in Fig. 4h, can also be reversibly switched (orange ⇄ yellow ⇄ green) under different bias conditions. The “EE” patterns exhibit a nine different colour array, while the previously reported conventional V_2_O_5_-based devices (configured as in Fig. 1a) only exhibit a three different colour array^13^. Figure 4i shows the change in the optical transmittance spectra of the large-area display under different bias conditions while maintaining an optical transparency of >40%. The reversible colour switching between orange and green colours of the display shows an optical transmission difference of 19% at 632.8 nm. The bar-coating method shows great potential for a large-scale, rapid, and inexpensive manufacturing capability, providing an efficient route to the commercialization of electrochromic indicators and tags. For high-resolution electrochromic displays, the inkjet printing method might be a potential approach because of its high-resolution features^14^.

### Zn–SVO electrochromic displays with broadened colour palettes

**Fig. 5:**
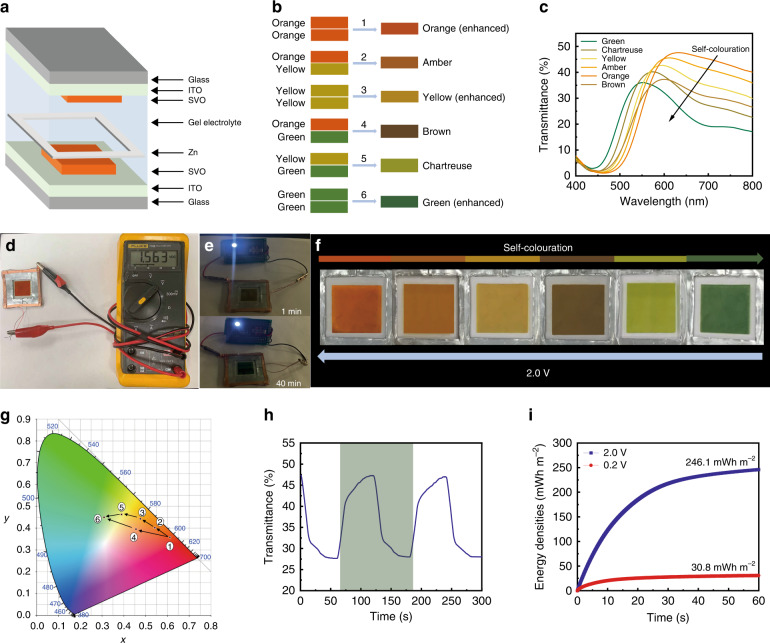
Design and performance of the Zn–SVO electrochromic displays with broadened colour palettes. **a** Schematic illustration of the Zn–SVO electrochromic display having six colours. **b** Schematic illustration of the colour overlay effect via the combination of orange, yellow and green colours. The upper colour code represents the colour of the top SVO electrode, while the lower colour code represents the colour of the bottom SVO electrode. **c** Visible-near infrared transmittance spectra of the display under different colour states. **d** Digital photograph of the display having an orange colour, showing an OCP of 1.56 V. **e** Digital photographs of a 0.5 V regulated LED powered by the Zn–SVO electrochromic display at 1 and 40 min. **f** Digital photographs of the Zn–SVO display showing six colours obtained through the colour overlay effect. **g** CIE colour coordinates of the Zn–SVO electrochromic display under different colour states. The six labelled numbers correspond to the six colours in (**c**). **h** Dynamic test of the display at 632.8 nm in the 0.2–2.0 V window. **i** Energy density comparison during the selected round-trip cycle (dark-green region) in (**h**).

As previously discussed, our newly established Zn-based electrochromic device enables a compelling colour overlay effect for electrochromic displays, which could significantly broaden the colour palettes of the displays. To implement such an innovative platform for multicolour Zn-based electrochromic displays, a Zn–SVO device (5 cm × 5 cm) was assembled and is schematically shown in Fig. 5a. The colour overlay effect is achieved through a combination of two SVO electrode segments to broaden the resultant colour palette. Such a device configuration is different from the configuration shown in Fig. 4g. To demonstrate the different colour array attained via the Zn-based electrochromic device, we assembled the device shown in Fig. 4g. Here, the device configuration in Fig. 5a is used to demonstrate the colour overlay of two SVO electrode segments. Since the two SVO electrode segments can be coloured and bleached independently, multiple colours can be achieved via the colour overlay effect of the three intrinsic colours (i.e., orange, yellow and green). Figure 5b illustrates the colour overlay effect obtained by superimposing the orange, yellow, and green colours. Since a single SVO electrode exhibits a three-colour behaviour (orange ⇄ yellow ⇄ green, Fig. 4c), the device can achieve six colours (i.e., orange, amber, yellow, brown, chartreuse and green). For example, the brown colour can be obtained via colour overlay of the top orange electrode and the bottom green electrode. Figure 5c shows the change in the optical transmittance of the electrochromic display under different colour states. There are four intermediate colours between the orange and green colours, which significantly broadens the colour palette of the display. The device maintains a semitransparency of >30%, which is a great advantage compared with the recently reported multicolour electrochromic displays using nanocavity structures^7,8^. The gradual colour switching from orange to green results in a 76 nm blueshift of the transmission peak from 627 to 551 nm while achieving a 20% optical contrast at 632.8 nm.

Interestingly, the orange-coloured display possesses an open-circuit potential (OCP) of 1.56 V (Fig. 5d) as it is in the fully charged state, which enables a self-colouration behaviour and energy retrieval functionality^17,34^. This OCP stems from the redox potential difference between the zinc foil and the SVO electrode, which provides the driving force that activates oxidation of Zn (i.e., stripping of Zn into the electrolyte) and reduction of the SVO film (i.e., intercalation of Zn^2+^ into SVO)^9^. Thus, the built-in voltage allows the display to switch its colour from orange to green (including the four intermediate colours) due to the reduction of the SVO film while powering an LED for more than 40 min (Fig. 5e). These vivid colours from the electrochromic display are shown in Fig. 5f. Conversely, the green-coloured display can be recovered to the orange colour via a charging process, wherein Zn is plated onto Zn foil and Zn^2+^ is extracted from the reduced SVO electrode. This charging process leads to bleaching of the SVO electrode. Figure 5g illustrates the corresponding chromaticity coordinates of the six colours within the CIE colour space. Such brilliant colour changes cause a remarkable shift of the CIE colour coordinates, forming a circular area from orange to green with different tints. The dynamic transmittance characteristics of the electrochromic display were evaluated in the 0.2–2.0 V window (Fig. 5h), where the response times are calculated to be 23.2 s for colouration and 34.8 s for bleaching. The orange colour indicates that the device is in the fully charged state, while the green colour indicates that the device is in the fully discharged state. Therefore, a colour change between the six displayed colours of the Zn–SVO electrochromic display can be triggered by lighting an LED. Furthermore, the amber, yellow, brown and chartreuse colours are intermediate colour states. As such, the response times of colour switching between these colours are faster than the values (23.2 s for colouration, 34.8 s for bleaching) shown in Fig. 5h. This device is also energy efficient. The energy densities during the dynamic switching between the orange state and the green state are shown in Fig. 5i, where 30.8 mWh m^−2^ is retrieved from the operating 246.1 mWh m^−2^.

## Discussion

In summary, the first demonstration of a transparent inorganic multicolour display, constructed by utilizing our newly established zinc-based electrochromic devices, was presented. The SVO electrode was fabricated via a bar-coating method with careful design of an eco-friendly SVO/cellulose paste. Such SVO electrodes deliver reversible colour switching (orange ⇄ yellow ⇄ green) associated with Zn^2+^ insertion (self-colouring/discharging) and extraction (bleaching/charging) while having a high optical transparency. The three intrinsic orange, yellow, and green colours were utilized as basic colours to develop multicolour Zn–SVO electrochromic displays via the colour overlay effect of two segments of the SVO electrodes. The constructed electrochromic display shows switching between the multiple colours (orange, amber, yellow, brown, chartreuse and green) and retrieves some of the consumed energy. These key properties mark a significant improvement over reported electrochromic displays, making the Zn–SVO electrochromic displays promising for switchable optical filters, electrochromic tuneable micro-optics and transparent displays. Our platform represents a new paradigm in electrochromic displays that can potentially facilitate new opportunities for the development of next-generation electrochromic displays.

## Materials and methods

### Materials

All the chemicals were of analytical grade and were used without further purification. Zinc sulfate heptahydrate (ZnSO_4_·7H_2_O, 99%), zinc foil (Zn, 99.9%), vanadium oxide (V_2_O_5_, 99%), sodium chloride (anhydrous, 99%) and polyvinyl alcohol (PVA, Mw ~130,000) were purchased from Sigma‐Aldrich. Cellulose was obtained from Alberta-Pacific Forest Industries Inc.

### Synthesis of sodium vanadium nanorods

Briefly, 100 g of commercial V_2_O_5_ powder was added into 1.5 L of a NaCl aqueous solution (2 M) at room temperature and stirred for 96 h to form a solution with a brownish suspension. Next, purification was conducted by adding distilled water, followed by centrifugation. The centrifugation process was repeated six times. Afterward, the product was diluted with distilled water to form a precursor solution (15 mg mL^−1^). Next, the precursor solution was sonicated in an ultrasonic bath until a clear SVO colloid was formed.

### Fabrication of electrodes

To meet the high viscosity requirement for the bar-coating method, 3 g of cellulose was added to 60 mL of the SVO colloid (15 mg mL^−1^) at 60 °C under stirring for 24 h. Prior to deposition, ITO-coated glass substrates were cleaned with ethanol and deionized (DI) water. Next, the SVO/cellulose paste was bar-coated onto an ITO glass substrate (2 cm × 5 cm), covering an effective area of 1 cm × 4 cm (see Fig. 3a for details). The SVO electrodes for the “EE” display were prepared by bar-coating SVO/cellulose paste onto an “EE” shaped cut-out mask on top of an ITO glass substrate (10 cm × 10 cm). The effective area of one “E” letter is 5 cm^2^, and the total effective area of the whole “EE” display is 10 cm^2^. The SVO electrodes for the display with the colour overlay effect were prepared by bar-coating SVO/cellulose paste onto a 2 cm × 2 cm cut-out square mask on top of an ITO glass substrate (5 cm × 5 cm). To remove the cellulose, all the bar-coated samples were annealed in air at 200 °C for 24 h.

### Assembly of electrochromic displays

A PVA-ZnSO_4_ gel was prepared by gradually adding 6 g of PVA to 60 mL of a ZnSO_4_ solution (0.5 M), which was stirred and heated in a water bath. The Zn–SVO electrochromic displays were constructed by sandwiching a thin Zn square frame between two pieces of SVO electrodes. The PVA-ZnSO_4_ gel was used as the electrolyte.

### Characterization

To analyse the composition and morphology of the samples, X-ray powder diffraction (XRD) (Bruker D8‐Advance) with Cu Kα‐radiation, X-ray photoelectron spectroscopy (XPS) (Kratos AXIS Ultra), transmission electron microscopy (TEM) (JEM-ARM200CF, JEOL), Fourier transform infrared spectroscopy (FTIR-iS50) and field emission scanning electron microscopy (FESEM) (Zeiss SIGMA FESEM, Germany) were used. Ex situ XPS measurements were conducted immediately after the samples were annealed, coloured and bleached. An Alpha-Step (IQ-W1-040) was used to reveal the thickness of the bar-coated SVO film. All electrochemical measurements were carried out using a Zahner electrochemical workstation (Zennium CIMPS-1). For the CV measurements of the SVO electrode in 1 M LiCl and 0.5 M ZnSO_4_, a three-electrode configuration was used with Pt wire and Ag/AgCl as counter and reference electrodes, respectively. Other electrochemical measurements were performed using a two-electrode configuration. The spectroscopy test for visible light transmittance spectra was conducted with an Ocean Optics USB4000 Spectrometer without subtracting the transmittance loss of ITO glass. Dynamic characterization of the SVO electrode was conducted by transmitting a helium-neon laser (632.8 nm) through the samples. A voltage was applied from a Zahner electrochemical workstation, and the photodiode output signal was collected with an oscilloscope. The response times were calculated based on the time required to achieve 90% of the maximum optical contrast. The International Commission on Illumination (CIE) 1931 colour space was used to demonstrate chromaticity coordinates.

## Supplementary information

Supplementary Information

## References

[1] Cao S (2019). A visible light-near-infrared dual-band smart window with internal energy storage. Joule.

[2] Zhang SL (2018). Al^3+^ intercalation/de-intercalation-enabled dual-band electrochromic smart windows with a high optical modulation, quick response and long cycle life. Energy Environ. Sci..

[3] Li HZ (2018). Nanohybridization of molybdenum oxide with tungsten molybdenum oxide nanowires for solution-processed fully reversible switching of energy storing smart windows. Nano Energy.

[4] Wang YY (2019). A multicolour bistable electronic shelf label based on intramolecular proton-coupled electron transfer. Nat. Mater..

[5] Xu T (2016). High-contrast and fast electrochromic switching enabled by plasmonics. Nat. Commun..

[6] Wade CR, Li MY, Dincǎ M (2013). Facile deposition of multicolored electrochromic metal-organic framework thin films. Angew. Chem. Int. Ed..

[7] Wang Z (2020). Towards full-colour tunability of inorganic electrochromic devices using ultracompact fabry-perot nanocavities. Nat. Commun..

[8] Hopmann E, Elezzabi AY (2020). Plasmochromic nanocavity dynamic light color switching. Nano Lett..

[9] Li HZ (2019). Rechargeable aqueous electrochromic batteries utilizing Ti-substituted tungsten molybdenum oxide based Zn^2+^ ion intercalation cathodes. Adv. Mater..

[10] Zhang SL (2019). Dual-band electrochromic devices with a transparent conductive capacitive charge-balancing anode. ACS Appl. Mater. Interfaces.

[11] Cao S (2018). Fluoride-assisted synthesis of plasmonic colloidal Ta-doped TiO_2_ nanocrystals for near-infrared and visible-light selective electrochromic modulation. Chem. Mater..

[12] Scherer MRJ (2012). Enhanced electrochromism in gyroid-structured vanadium pentoxide. Adv. Mater..

[13] Wei D (2012). A nanostructured electrochromic supercapacitor. Nano Lett..

[14] Costa C (2012). Electrochromic properties of inkjet printed vanadium oxide gel on flexible polyethylene terephthalate/indium tin oxide electrodes. ACS Appl. Mater. Interfaces.

[15] Zhao GF (2019). A multicolor electrochromic film based on a SnO_2_/V_2_O_5_ core/shell structure for adaptive camouflage. J. Mater. Chem. C..

[16] Li HZ, Firby CJ, Elezzabi AY (2019). Rechargeable aqueous hybrid Zn^2+^/Al^3+^ electrochromic batteries. Joule.

[17] Li HZ, Elezzabi AY (2020). Simultaneously enabling dynamic transparency control and electrical energy storage via electrochromism. Nanoscale Horiz..

[18] He P (2018). Sodium ion stabilized vanadium oxide nanowire cathode for high-performance zinc-ion batteries. Adv. Energy Mater..

[19] Mjejri I (2018). Crystallized V_2_O_5_ as oxidized phase for unexpected multicolor electrochromism in V_2_O_3_ thick film. ACS Appl. Energy Mater..

[20] Zhang W (2020). Electrochromic battery displays with energy retrieval functions using solution-processable colloidal vanadium oxide nanoparticles. Adv. Optical Mater..

[21] Chen R (2019). Sequential solution polymerization of poly(3,4-ethylenedioxythiophene) using V_2_O_5_ as oxidant for flexible touch sensors. iScience.

[22] Khim D (2013). Simple bar-coating process for large-area, high-performance organic field-effect transistors and ambipolar complementary integrated circuits. Adv. Mater..

[23] Margoni MM (2018). Hydrothermally grown nano and microstructured V_2_O_5_ thin films for electrochromic application. Appl. Surf. Sci..

[24] Wan F (2018). Aqueous rechargeable zinc/sodium vanadate batteries with enhanced performance from simultaneous insertion of dual carriers. Nat. Commun..

[25] Shazali NAH (2019). Characterization and cellular internalization of spherical cellulose nanocrystals (CNC) into normal and cancerous fibroblasts. Materials.

[26] Onbattuvelli VP (2020). Structure and thermal stability of cellulose nanocrystal/polysulfone nanocomposites. Mater. Today Commun..

[27] Jordan JH (2019). Extraction and characterization of nanocellulose crystals from cotton gin motes and cotton gin waste. Cellulose.

[28] Silversmit G (2004). Determination of the V2p XPS binding energies for different vanadium oxidation states (V^5+^ to V^0+^). J. Electron Spectrosc. Relat. Phenom..

[29] Li HZ, McRae L, Elezzabi AY (2018). Solution-processed interfacial PEDOT: PSS assembly into porous tungsten molybdenum oxide nanocomposite films for electrochromic applications. ACS Appl. Mater. Interfaces.

[30] Kang WB (2014). Green synthesis of nanobelt-membrane hybrid structured vanadium oxide with high electrochromic contrast. J. Mater. Chem. C..

[31] Tong ZQ (2016). Annealing synthesis of coralline V_2_O_5_ nanorod architecture for multicolor energy-efficient electrochromic device. Sol. Energy Mater. Sol. Cells.

[32] Li HZ (2017). Solution-processed porous tungsten molybdenum oxide electrodes for energy storage smart windows. Adv. Mater. Technol..

[33] Cai GF (2013). One-step fabrication of nanostructured NiO films from deep eutectic solvent with enhanced electrochromic performance. J. Mater. Chem. A.

[34] Zhang SL (2020). Overcoming the technical challenges in Al anode-based electrochromic energy storage windows. Small Methods.

