# Supplementary material for: Transparent inorganic multicolour displays enabled by zinc-based electrochromic devices
Source: Light Sci Appl. 2020 Jul 14;9:121. doi: 10.1038/s41377-020-00366-9 (PMC7360616; doi:10.1038/s41377-020-00366-9)
Supplement: Supplementary file 1 — Supplementary Information [file 41377_2020_366_MOESM1_ESM.docx]

**Supplementary Information**

**Transparent inorganic multicolour displays enabled by zinc-based electrochromic devices**

Wu Zhang^1^, Haizeng Li^1*^, William W. Yu^2^, Abdulhakem Y. Elezzabi^1*^

^1^Ultrafast Optics and Nanophotonics Laboratory, Department of Electrical and Computer Engineering, University of Alberta, Edmonton, Alberta, T6G 2V4, Canada

^2^Department of Chemistry and Physics, Louisiana State University, Shreveport, Louisiana 71115, United States

^*^ To whom correspondence should be addressed. Email: [haizeng@ualberta.ca](mailto:haizeng@ualberta.ca) (Haizeng Li), [elezzabi@ualberta.ca](mailto:elezzabi@ualberta.ca) (A.Y. Elezzabi)

Keywords: Sodium Vanadium Oxide, Bar-coating Method, Multicolour Electrochromic Display, Colour Overlay


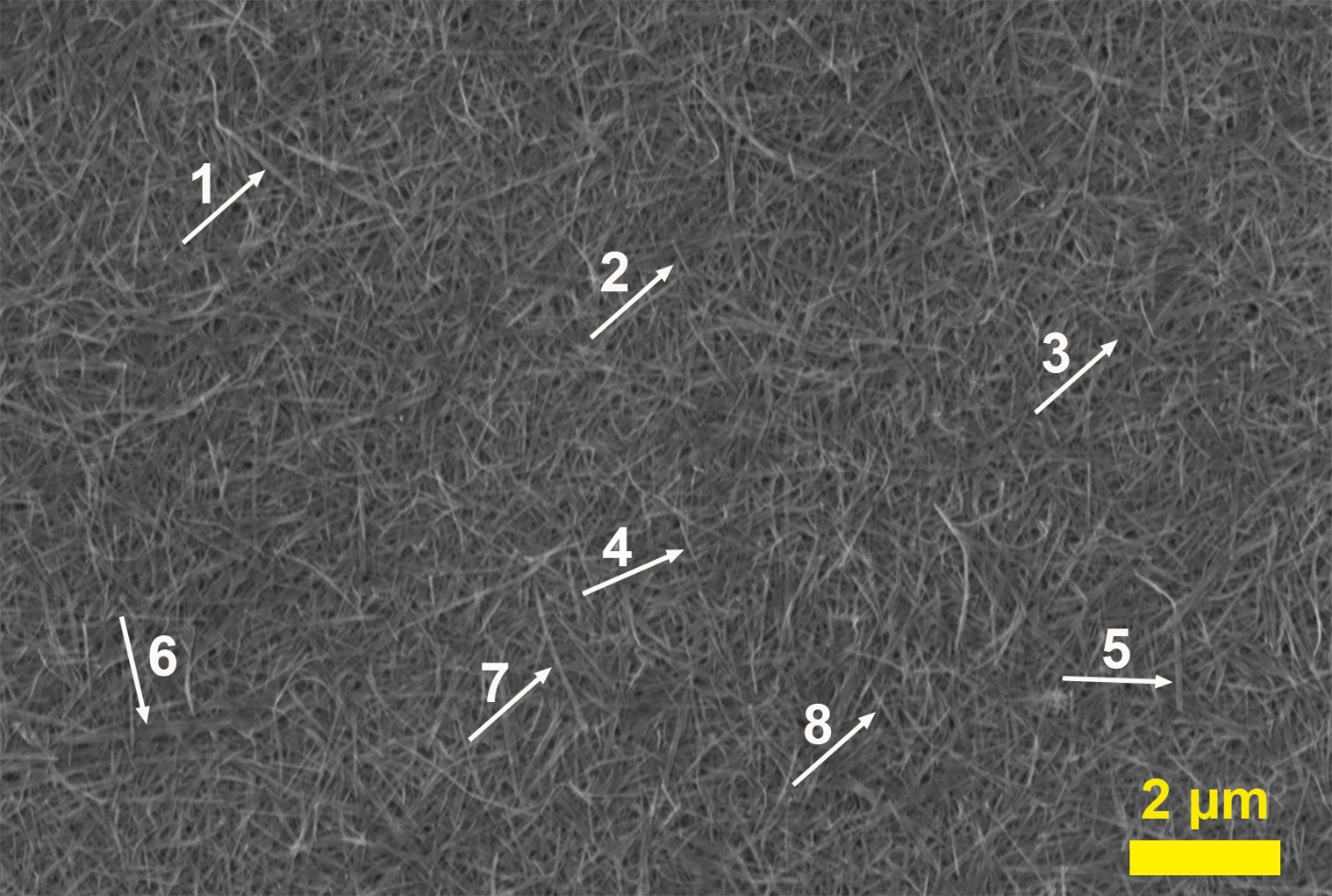


**Supplementary Figure S1.** FESEM image of the SVO nanorods.

**Supplementary Table S1**. Diameter and length of the SVO nanorods.

| **Sample** | **Diameter (nm)** | | **Length (μm)** |
| --- | --- | --- | --- |
| 1 | 50 | 1.4 | |
| 2  3  4 | 20  30  20 | 0.5  1.7  0.6 | |
| 5  6  7  8  **Range** | 50  60  40  30  **20-60** | 1.3  2.0  1.9  0.8  **0.5-2.0** | |

Supplementary Figure S1 shows the FESEM image of the SVO nanorods. Eight SVO nanorods with a specific diameter and length are shown in Supplementary Table S1. The SVO nanorods range from 0.5-2.0 μm in length and 20–60 nm in diameter.


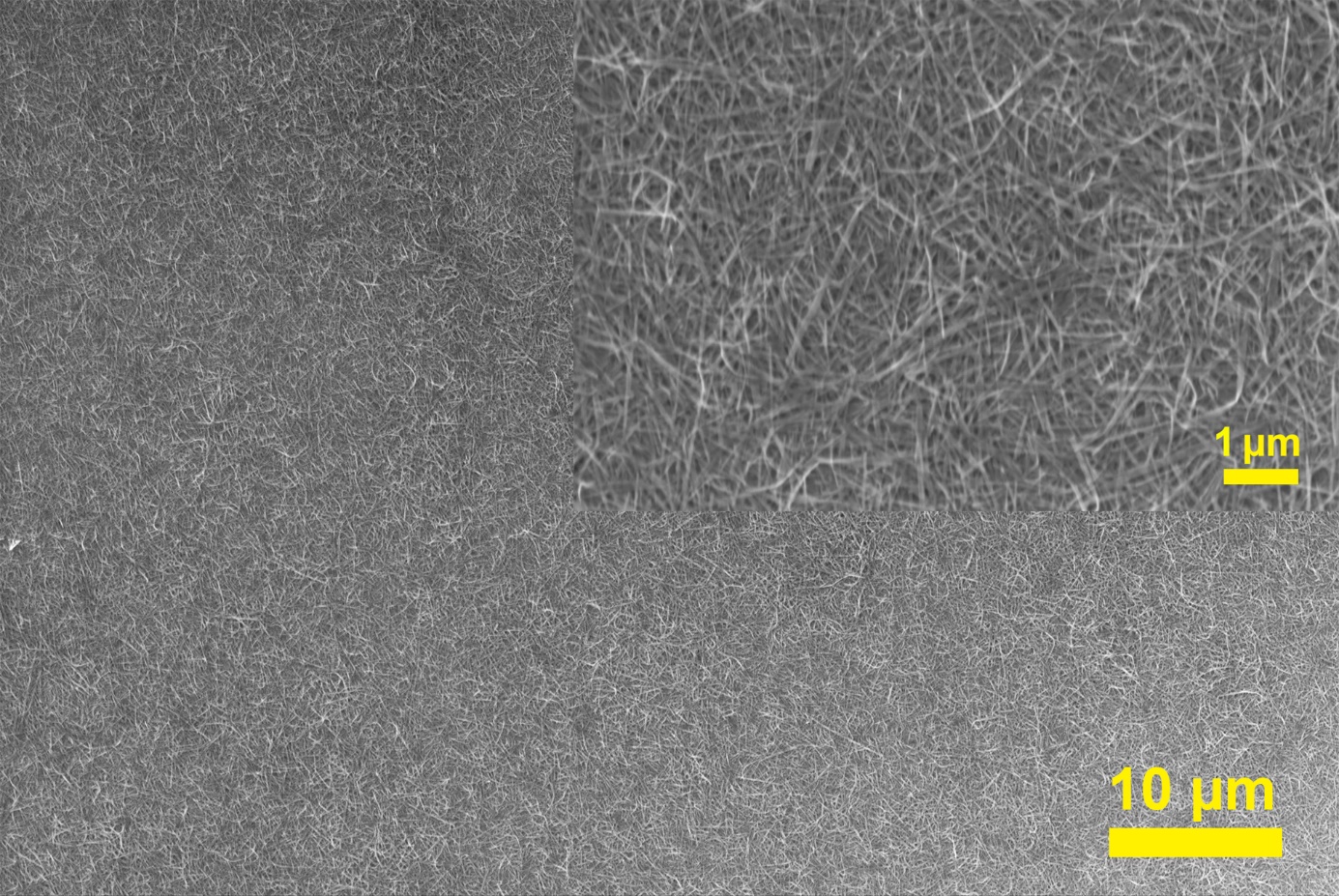


**Supplementary Figure S2.** FESEM images of the bar-coated and annealed SVO films.

Supplementary Figure S2 shows FESEM images of the bar-coated SVO films after the annealing process. The homogenous nanorods morphology is consistent with the morphology of SVO nanorods shown in Figs. 2c, d.





**Supplementary Figure S3.** Cyclic voltammetry (CV) measurement of SVO electrodes performed at 50 mV s^-1^ in 1 M LiCl electrolyte and 0.5 M ZnSO_4_ electrolyte.

The CV curves of the SVO electrodes in different electrolytes (1 M LiCl and 0.5 M ZnSO_4_) are shown in Supplementary Fig. S3. The reduction and oxidation peaks (around 0.10/0.56 V) suggest the intercalation and deintercalation processes. The CV curve of the SVO cathode, measured in the ZnSO_4_ electrolyte, exhibits 1.4 times higher capacity compared to that tested in LiCl. This indicates that the SVO cathode is more electrochemically active towards the Zn^2+^. The underlying process may be attributed to the large radius of hydrated sodium ions that enlarge interlayer space and voids^1^.


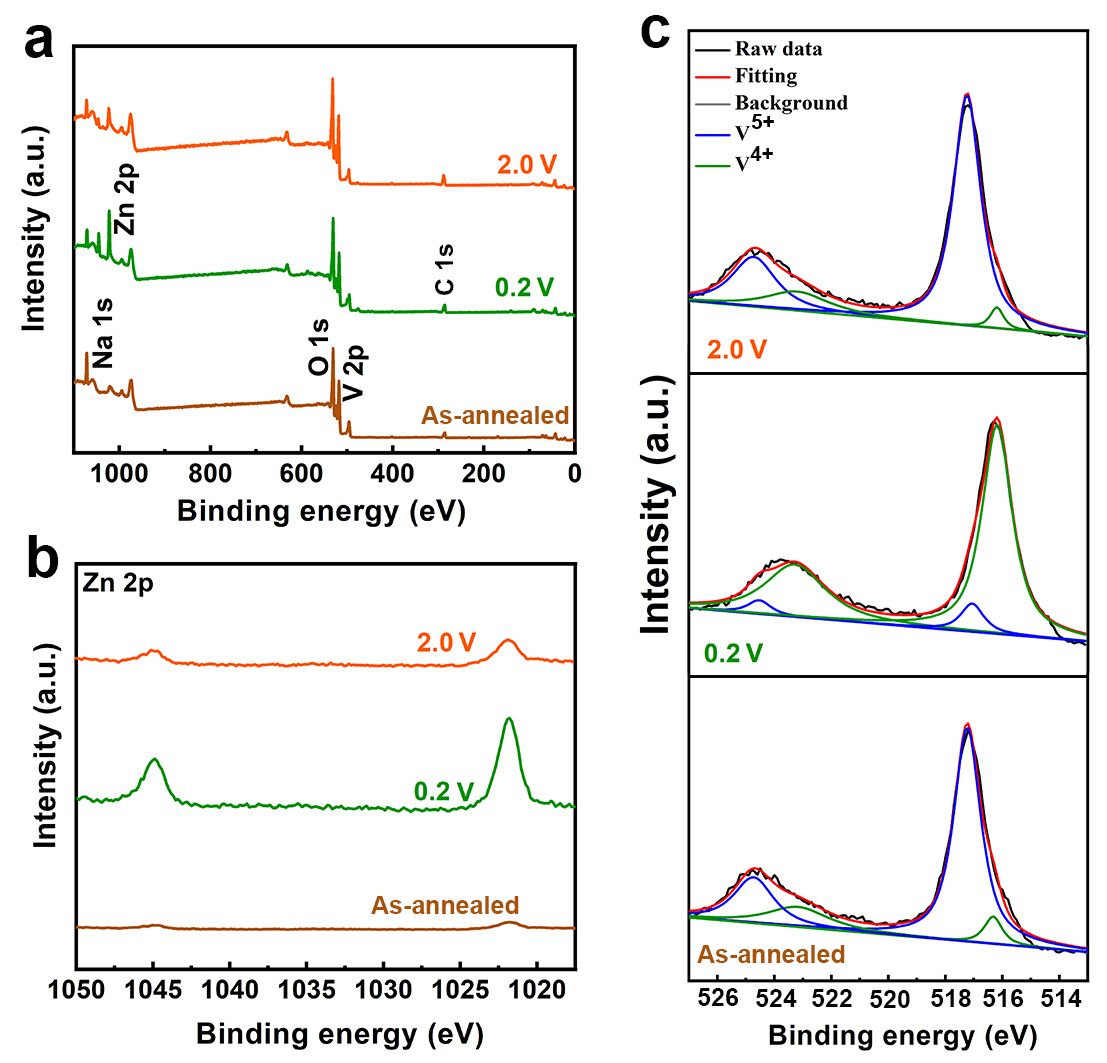


**Supplementary Figure S4.** The ex-situ XPS survey spectra of the SVO electrode during discharging/charging: (**a**) Full survey (**b**) Zn 2p, and (**c**) V 2p*.*

To analyze the operation of the Zn-SVO electrochromic display, ex-situ X-ray photoelectron spectroscopy (XPS) measurements were carried out to evaluate the valence state of the V accompanied by Zn^2+^ insertion/extraction. As illustrated in Supplementary Fig. S4a, the ex-situ XPS full survey spectra of the SVO electrode indicates the presence of Na, V, and O elements, without other impurities (excluding C). The presence of Zn 2p peaks in the tinted SVO film (0.2 V) confirms the insertion of Zn^2+^. The weak intensities of Zn 2p peaks in the bleached SVO film (2.0 V) indicate that most of the Zn^2+^ cations are extracted from the SVO electrode. The presence of Zn^2+^ residue in the electrode is due to the intercalated Zn^2+^ cations are trapped at the “dead Zn^2+^ sites”^2^. The trapped Zn^2+^ ions can be extracted from the dead Zn^2+^ sites by applying a high current under a stable voltage window of the electrolyte for real applications^3^. These features are further confirmed by the high-resolution Zn 2p core-level XPS spectra (Supplementary Fig. S4b). Supplementary Figure S4c depicts the high-resolution V 2p core-level XPS spectra of the SVO film under different states (charging/discharging). As expected, the intercalation/deintercalation of Zn^2+^ would induce the valence state change of the V. The most intense doublet peaks, located at 517.2 and 524.6 eV, are assigned to the V^5+ 4,5^. Another pair of peaks, centered at 516.2 and 523.2 eV, are corresponded to the V^4+^. The atomic ratio of V^4+^/V^5+^ for the as-annealed SVO film is estimated to be 0.12 (Supplementary Table S2). During the self-colouration process (0.2 V), the high valence state V^5+^ was partially reduced to V^4+^ and the atomic ratio of V^4+^/V^5+^ increased to 6.69 (Supplementary Table S2). In another part, the V^4+^/V^5+^ atomic ratio of the bleached film (2.0 V) is estimated to be 0.10 (Supplementary Table S2). The switch of V^4+^/V^5+^ ratio during discharging/charging induces the colour switch of such Zn-SVO electrochromic device.





**Supplementary Figure S5.** Colouration Efficiency (CE) of the SVO film.

The colouration efficiency, defined as the change in optical density (ΔOD) per unit of charge intercalated into the electrochromic layer at a particular wavelength^6^, is calculated to be 61.2 cm^2^ C^-1^ for the SVO film.


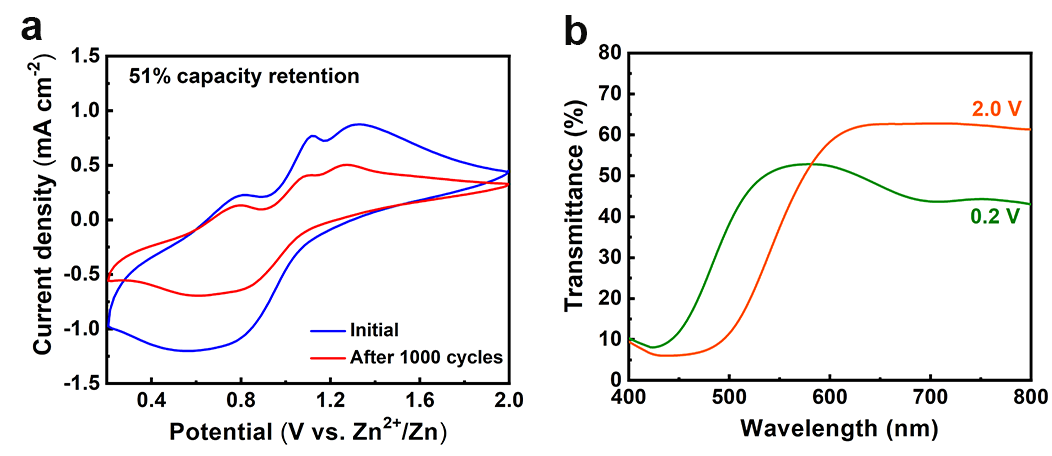


**Supplementary Figure S6.** Cycle performance of the SVO electrode: **a** CV measurement performed at 50 mV s^-1^, **b** Visible-near infrared transmittance spectra after 1000 cycles measured at 0.2 V and 2.0 V*.*

The cycling measurements for extended cycles were conducted by the cyclic voltammetric scanning between 0.2 V and 2.0 V at a scan rate of 50 mV s^-1^. As shown in Supplementary Fig. S6, the SVO electrode retains 51% of its initial capacity and has a 13% optical contrast after 1000 CV cycles. This degradation can be attributed to the expansion and exfoliation of the SVO film, and electrochemical grinding phenomena during cycling^7^.

**Supplementary Table S2**. Atomic ratio of V^4+^ and V^5+^ in the as-deposited, annealed for 30 mins, annealed for 24 h, reduced (0.2 V) and oxidized (2.0 V) SVO film.

| **Sample** | **Atomic percentage of V in different valence states** | | **V^4+^/V^5+^** | | |
| --- | --- | --- | --- | --- | --- |
|  | **V^4+^** | **V^5+^** | |  |  |
| As-deposited | 0.00 | 1.00 | | **0.00** |  |
| Annealed for 30 mins  Annealed for 24 h  Reduced (0.2 V) | 0.29  0.11  0.87 | 0.71  0.89  0.13 | | **0.41**  **0.12**  **6.69** |  |
| Oxidized (2.0 V) | 0.09 | 0.91 | | **0.10** |  |

**Supplementary References**

1. Ming, F. *et al.* Layered Mg_x_V_2_O_5_·nH_2_O as cathode material for high-performance aqueous zinc ion batteries. *ACS Energy Lett.* **3**, 2602-2609 (2018).

2. He, P. *et al.* High-performance aqueous zinc–ion battery based on layered H_2_V_3_O_8_ nanowire cathode. *Small* **13**, 1702551 (2017).

3. Wen, R. T., Granqvist, C. G. & Niklasson, G. A. Eliminating degradation and uncovering ion-trapping dynamics in electrochromic WO_3_ thin films. *Nat. Mater.* **14**, 996-1001 (2015).

4. Silversmit, G., Depla, D., Poelman, H., Marin, G. B. & De Gryse, R. Determination of the V2p XPS binding energies for different vanadium oxidation states (V^5+^ to V^0+^). *J. Electron Spectros. Relat. Phenomena* **135**, 167-175 (2004).

5. Zhang, W., Li, H., Al-Hussein, M. & Elezzabi, A. Y. Electrochromic battery displays with energy retrieval functions using solution-processable colloidal vanadium oxide nanoparticles. *Adv. Opt. Mater.* **8**, 1901224 (2020).

6. Heo, S. *et al.* Enhanced coloration efficiency of electrochromic tungsten oxide nanorods by site selective occupation of sodium ions. *Nano Lett.* **20**, 2072-2079 (2020).

7. Senguttuvan, P. *et al.* A high power rechargeable nonaqueous multivalent Zn/V_2_O_5_ battery. *Adv. Energy Mater.* **6**, 1600826 (2016).
